# Changing Academic Medicine: Strategies Used by Academic Leaders of Integrative Medicine—A Qualitative Study

**DOI:** 10.1155/2012/652546

**Published:** 2012-10-11

**Authors:** Claudia M. Witt, Christine Holmberg

**Affiliations:** ^1^Institute for Social Medicine, Epidemiology, and Health Economics, Charité University Medical Center Berlin, Luisenstraße 57, 10117 Berlin, Germany; ^2^Center for Integrative Medicine, School of Medicine, University of Maryland, Baltimore, MD 27207, USA; ^3^Berlin School of Public Health, Charité University Medical Center Berlin, Seestraße 73, 13347 Berlin, Germany

## Abstract

In Western countries, complementary and alternative medicine (CAM) is more and more provided by practitioners and family doctors. To base this reality of health care provision on an evidence-base, academic medicine needs to be included in the development. In the study we aimed to gain information on a structured approach to include CAM in academic health centers. We conducted a semistructured interview study with leading experts of integrative medicine to analyze strategies of existing academic institutions of integrative medicine. The study sample consisted of a purposive sample of ten leaders that have successfully integrated CAM into medical schools in the USA, Great Britain, and Germany and the Director of the National Center for Alternative and Complementary Medicine. Analysis was based on content analysis. The prerequisite to foster change in academic medicine was a strong educational and professional background in academic medicine and research methodologies. With such a skill set, the interviewees identified a series of strategies to align themselves with colleagues from conventional medicine, such as creating common goals, networking, and establishing well-functioning research teams. In addition, there must be a vision of what should be needed to be at the center of all efforts in order to implement successful change.

## 1. Introduction

Complementary and alternative medicine (CAM) use is a major factor in the informal health care system in Western countries. According to the 2007 National Health Survey, nearly 40% of Americans used CAM within the last 12 months [1] and they spent $33.9 billion out of pocket for CAM [2]. In the United Kingdom (UK), a 2001 survey showed that 28% of the population has used CAM in the last 12 months [3]. Half of all general practitioners in the UK offered at least some CAM service to their patients [4]. Finally, in Germany, 70% of women and 54% of men had used at least one CAM treatment during a 12-month period [5]. Comparable to UK rates, in Germany, 60% of general practitioners offer some kind of CAM treatment [6]. However, international comparisons are difficult, for example, natural products are regulated very differently; some products that are regulated as dietary supplements in the US are regulated as drugs in Germany.

The high rate of CAM provision by general practitioners and the high prevalence of CAM use in society influenced academic medicine. To ensure high quality CAM provision, it is important that academic medicine considers a structured approach of introducing CAM into their curricula, research agendas, and model of health care delivery. Academic health centers are a driving force for innovation in health care and have a responsibility to high quality education and research. Since CAM is increasingly offered in conventional medicine settings, it is crucial that the evidence is created to provide sound information on CAM to providers and to include well-founded knowledge on CAM into medical curricula in order to achieve high-quality integrative medicine. 

Since the establishment of the National Center for Complementary and Alternative Medicine (NCCAM) as part of the National Institutes of Health (NIH) in 1998, medical research in the US has begun to emphasize high-quality and evidence-based assessment of the value of complementary therapies. Similarly, the European Union has started to fund CAM research in recent years. Thus, the move of CAM into academic medicine is currently under way and has already been introduced successfully in some academic centers across Germany, the US, and the UK. 

In the US, the establishment of CAM research as part of the NIH was in part fueled by an article published by Eisenberg et al. in the 1990s that demonstrated the frequent use of CAM in mainstream US society [7]. The article drew attention to the increasing need for research into CAM in order to evaluate CAM therapies for their safety, efficacy (or lack thereof) and mechanism. Particularly, the article highlighted the need to focus research on negative interactions between conventional medical treatments and CAM interventions used, and often unreported, by patients. The decision to establish the NCCAM increased the recognition of CAM research and facilitated its integration into conventional medicine. Indeed some leading, and highly innovative academic institutions in the US, Germany and the UK have established what are now, successful integrative centers at medical schools, the birthplace of scientific medicine. 

In this paper, we aim to provide information on a structured approach to establish CAM departments in academic health centers. An increase of such centers will be critical to support the current changes in health care provision and in patients' expectations towards and role in health care delivery. Academic institutions should be on the forefront of these developments. We use the most prominent departments of integrative medicine as exemplars in our study. 

A previous qualitative study has provided interesting details about research, education, clinical care, and administration of ten leading academic North American integrative medical centers [8]. Another study focused on factors that enable the establishment of integrative medicine in clinics [9]. In order to determine a structured approach towards the integration of CAM research and curricula in medical schools, this study analyzed the trajectory of exemplary integrative medicine departments in the top international academic health centers. What were the factors that made the successful integration of CAM into medical schools possible? In what types of structures could integrative medicine be established? Based on the analysis of the processes and factors that enabled the integration of CAM into existing, leading institutions, more general strategies were developed.

## 2. Methods

### 2.1. Study Design and Methods

The study is a semistructured qualitative interview study [10]. The interview guideline was developed by both authors based on their knowledge of the field of integrative medicine and the aim of the study. All interviews were conducted by one interviewer trained in qualitative methods (CW) from August 2010 to March 2011.

### 2.2. Study Sample

The study sample consists of a purposive sample of leaders that have introduced CAM research and curricula into medical schools in the USA, Great Britain, and Germany. Selection criteria included at least 10 years' experience with integrative medicine at medical schools, international reputation in integrative medicine, and visibility on international congresses and in academic associations. All interviewees were head or director of a center/clinic or program. In one center (Berlin) two experts were interviewed one with a research background and the other one with an additional strong clinical background. In addition to those selected with the aforementioned criteria, the director of the National Center for Alternative and Complementary Medicine (NCCAM) was included in the study sample. NCCAM is a driving force in establishing rigorous research in the field of CAM. Since it is the purpose of this paper to study strategies to bring CAM into academic centers, the evidence-base plays an important role and the perspective of NCCAM is of relevance for the study. For more information about the centers represented see Table 1.

Recruitment into the study continued until no additional information was received [11, 12]. All contacted persons agreed to participate in the study. Interviews were conducted face-to-face. 

Written informed consent was provided by all of the interview partners. To obscure the identification of interview partners in the results, we will not disclose personal characteristics such as gender, age, or country of residence.

### 2.3. Analysis

The interviews were tape-recorded and transcribed. Analysis followed a grounded theory approach [12] assisted by the qualitative computer software program maxqda [13]. Coding took place in several rounds. First, the themes of the interview guide were used to organize the materials and provide the initial codes. Then, each segment was analyzed according to themes present. Finally, categories that arose during analysis were bundled into core categories. A set of strategies and requirements were extracted from the transcripts that made it possible to establish integrative medicine in academic health centers. Analysis and results were regularly discussed in the research team and in a qualitative research group to ensure intersubjectivity and grounding of results in the material [12].

## 3. Results 

### 3.1. The Sample

The sample consisted of eight medical doctors (M.D.) and two Ph.D. holders. All participants were heads of centers or programs at medical schools, with one exception: the director of NCCAM who brought in governmental perspectives with a strong focus on rigorous research. Except for two, the interviewed heads of the centers and programs were also the founders of the center or program. Both Ph.D. holders became interested in the integration of CAM into regular medical research and curricula as a result of their professional and teaching experience. Two of the M.D. holders explicitly stated that they entered the medical field to integrate other medical systems into conventional medicine. Of the remaining six physicians, three became aware of CAM through the experience of treating patients with chronic and complex conditions in their clinical practices. Two of the physicians had attended courses in acupuncture, homeopathy, and other CAM modalities while they were medical students. Finally, one M.D. holder became interested in researching CAM after personally experiencing the positive effects of acupuncture. 

### 3.2. Important for Establishment of a New Academic Center for Integrative Medicine

#### 3.2.1. Requirements

**Strong Academic Background**

All of the interviewees were educated and trained in an established research field or in conventional medicine, mostly in prestigious academic institutions. The interviewees identified such a background as a critical factor that enabled them to establish integrative medicine research and curricula in academic centers. Education was discussed as an influential aspect of professional growth as well as the foundation for quality research agendas. Those who entered medical school with the intention of integrating CAM into their work saw the need to establish credentials in conventional medicine to be accepted in the academic medical community.

**Contribution to Research**

Some of the experts began their academic career with research on CAM with neither public funding nor much interest from others. Such initial studies became crucially important to establish further and funded research in CAM. For example, the interviewees and other CAM research experts used these studies to argue for larger scale and more rigorous research on CAM interventions to establish its safety and efficacy. This early research conducted with scientific expertise had interesting findings and proved to be an entrance into academic medicine.

**Clarity of Focus**

The focus on a personal goal, be it patient-orientation, education-orientation, or research-orientation, was stressed in several interviews. The need to have a vision and keep that vision at the center of all efforts and activities was seen as absolutely necessary to establish integrative medicine centers. The clear focus and orientation towards a goal was considered vital to the success of their work. Similarly most expressed a passion and enthusiasm associated with their respective goals.

#### 3.2.2. Strategies

In the narratives, the interviewees presented their success in establishing integrative medicine research and curricula in academic medicine; several strategies could be discerned that the experts used, intentionally and unintentionally, which made the success possible.

**Creating Common Goals**

For some interviewees, the introduction of CAM into academic health centers was aided by creating common goals with the dean or other department heads. For example, interest in evidence-based and comprehensive treatment options provided the foundation for collaboration with conventional medicine professionals. Similarly, an emphasis on educating students to become excellent medical doctors was a goal that different members of the faculty agreed upon and saw CAM as an aspect of such an education. Lastly, interviewees highlighted that the provision of CAM interventions could be aligned with economic interests of the health center as it would increase possibilities for marketing and revenue.

**Utilizing Preexisting Frameworks**

The experts used their intimate knowledge of the medical schools and research entities they were working in to connect their research interests to already existing research and initiatives at the clinic, such as an existing focus on prevention and well-being or pain management. Others highlighted the closeness of mainstream medical therapies in cardiovascular diseases with CAM, for example, stress reduction and nutrition guidance. In fact, some of the experts also transitioned into CAM research as a result of findings in their research such as placebo effects and nonpharmacological approaches to health.

Interviewees also mentioned institutional characteristics that proved advantageous to establishing integrative medicine centers. A preexisting focus on wellness, institutional diversity in leadership, and/or programs in “cultural competency” made certain institutions more likely to be responsive to integrating CAM research and curricula.

**Networking**

The importance of networking was evident in all interviews, both among peers as well as among the scientific and medical community at large. All reported that they maintained strong networks within the CAM research community. As CAM remains a marginal area of medical research, active engagement and mentorship among colleagues was seen as a crucial aspect of community building by the interviewees.

Similarly, networking across different disciplines and world views was a factor in establishing integrative centers. Some of the interviewees made a point of visiting all clinic directors to engage them in their endeavors and gain their support for the centers when they began their work.

**Team-Building**

In line with good networking, the experts attributed their successful integration of CAM to team-building and team-spirit. Some interviewees specifically stressed the necessity of good research teams. Good was defined in several ways: (1) a team that shared a vision, (2) a team that consisted of highly qualified scientists and clinicians, (3) a team that would enable the leaders to do the networking necessary and still produce great research, and (4) a team with members that were able to generate research funding.

**Figure 1:**
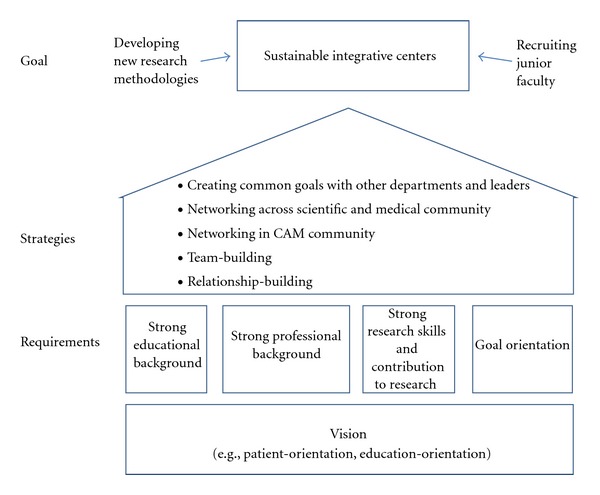
Important factors for the establishment and long-term sustainability of integrative centers.

For some, the success of integration was not only based on the establishment of a good research team in their respective academic health center. It also required interdisciplinary cooperation and expanded teams across various universities including members who were critical of CAM and integrative medicine (Figure 1).

### 3.3. Important for Sustainability

#### 3.3.1. General

Interviewees acknowledged additional factors that proved necessary to sustain long-term integration of CAM research and curricula in academic medical centers. Such factors included the treatment and research successes of the centers, the ability to generate funds continuously, and finally working effectively with media to maximize economic advantages of integrating CAM into clinics. Some saw the inclusion of evidence-based CAM into treatment guidelines as crucial for the sustainability of integrative medicine. Thus, the importance of successful establishment of research could not be overestimated. 

#### 3.3.2. Development of Appropriate Research Models

All of the experts saw the need to improve and develop scientifically rigorous methods of determining the efficacy and effectiveness of CAM. While there was some discrepancy between perspectives on whether traditional methods of evidence-based research were entirely possible, specifically if such methodological approaches to establishing efficacy may in fact destroy some of the healing qualities of CAM, all experts agreed that such an evidence base was needed and all experts worked actively towards this goal. At the same time, on the expert and funders side, the need to develop new methodologies of evidence creation in the future was addressed because the double-blind trial is not feasible with some CAM methods (e.g., mind body medicine). Thus, to move integrative medicine forward and to create the best possible medicine available, a focus on methods development is tantamount to evidence creation. The director of NCCAM highlighted the role of NCCAM as leader in research and research support. She saw the development of research methods that fit rigorous scientific standards and take into account the specificities of CAM as one of the most important tasks for the future.

#### 3.3.3. Recruitment and Mentorship of Junior Faculty

To establish such long-term research agendas, the experts agreed that junior faculty need to be attracted to the field of integrative medicine. To achieve such a long-term integration of CAM in medical schools, the interviewees made recommendations on what may be important for those who follow them (Table 2).

Considering the hard work it took to change academic medicine, the experts maintained that it requires a special kind of person and a unique set of skills to enable integration. Characteristics they suggested future leaders should possess included being indebted to a theme with passion, curiosity, and persistence that is not solely fueled by career interests. Interviewees stressed the need for interpersonal skills that integrate and enable different disciplines and fields to collaborate. Finally, a critical mind and skepticism were traits the interviewees thought leaders in the field needed since the foundation of sustainability was seen to be research.

## 4. Discussion

The integration of complementary and alternative medicine (CAM) research and curricula in academic medical centers necessitates a structured and systematic approach. The requirements and strategies highlighted by the expert interviewees include qualities of leadership and strategies for implementation, as well as requirements for further development and sustainability of the field.

Overall, a standardized and rigorous scientific approach is deemed essential in the effort to integrate CAM into academic health centers. It was through science, or a categorically scientific research model, that public and institutional support was created. The interviewees' knowledge and skills in such research methodology earned them respect within the larger academic medicine community and bolstered credibility for investors. Polich et al. [14] have shown the importance to “act a little more scientific” when working in integrative medicine. In their study on the fluid boundaries between CAM and conventional medicine, they found that CAM scientists strategically used methodological stringency to deal with perceived peer-review bias. In this study, we found that the methodological stringency was also used as a means to integrate CAM into academic medicine.

The general trend within academia towards evidence-based medicine [15–17] paved the way for integrative research agendas and curricula in academic centers. Firstly, aligning interest in evidenced-based treatments enabled CAM researchers to collaborate with colleagues in other areas of academic medicine using a shared methodological approach. Secondly, having a common “scientific” foundation allowed for an increased openness toward complementary medical systems, ideologies, and philosophies. Thirdly, evidence-based medicine could be used by the experts to counter the arguments against CAM from within academic medicine [18]. Evidence-based medicine was therefore the chosen avenue for integration and those who brought about such change emphasized the necessity of high quality research skills. 

The experts stressed the need for a clear vision or a goal through which all efforts should be focused. The vision that united most of the interviewees was patient-centeredness, as well as the need to provide treatment that is suitable for the individual patient. Patient-centeredness and personalized medicine are two major topics in the current discourse on treatment models in conventional health care [19–23]. Personalized medicine is generally understood in genetic terms [24] and encompasses only pharmacological approaches to treatment. It may, however, provide a potential avenue for integration of CAM research and subsequent broadening of the term.

An individualized approach, focus on the patient's specific treatment needs, and involvement of the patient in his or her care are hallmarks of integrative medicine and could thus be aligned with the above mentioned trends prevalent in western health care systems today [21–23]. The sample for this study is a highly selected group of leaders in the field of integrative medicine; therefore, the strategies presented may not represent all requirements and strategies for integrating CAM in academic centers. The research question of how integrative medicine can become part of academic medicine necessitated a qualitative design [10].

## 5. Conclusion

The study results show structural approaches to the integration of CAM research and curricula in academic medicine through collaboration, utilizing existing frameworks, and adopting a rigorous scientific approach. Integration required skillful negotiations, open-minded peers, like-minded researchers, and an economic environment that made it beneficial for hospitals and research centers to include patients' preferences in their own care delivery. This combination of personal skills paired with congruent advancements in academic medicine, economy, and society enabled the establishment of eight highly successful academic centres of integrative medicine that stand at the forefront of academic medicine. The strategies presented and analyzed here may provide other academic medical centers with the tools to broaden quality-oriented outcomes and incorporate patient-centeredness and personalized medicine with the integration of CAM research and curricula. 

## Figures and Tables

**Table 1:** Centers and programs that were represented by the interview partners.

Center	Established	Research	Teaching	Outpatient clinic	Inpatient clinic
USA					

Center for Integrative Medicine University of Maryland School of Medicine	1991	yes	Undergraduate Postgraduate	yes	Consulting service
Osher Center for Integrative Medicine, USFC, San Francisco	1997	yes	Undergraduate Postgraduate	yes	no
National Center for Complementary and Alternative Medicine (NCCAM) at NIH	1998	yes	Postgraduate	no	no
Harvard Medical School Osher Research Center, The Division for Research and Education in Complementary and Integrative Medical Therapies	2000	yes	Postgraduate	yes	no
Education Program in CAM and Integrative Medicine, Georgetown University Medical Center, Washington, D.C.	2001	yes	Graduate Postgraduate	no	no
The Institute for Complementary and Alternative Medicine, University of Medicine and Dentistry for New Jersey	2002	yes	Graduate Postgraduate	no	no

UK					

Complementary and Integrated Medicine Research Unit, University of Southampton	1995	yes	Graduate Postgraduate	no	no

Germany					

Institute for Social Medicine, Epidemiology and Health Economics, Charité University Medical Center Berlin*	1998	yes	Undergraduate Postgraduate	yes	yes
Chair for Complementary and Integrative Medicine, University of Duisburg-Essen, Germany, Knappschafts-Krankenhaus Essen-Mitte	1999	yes	Undergraduate Postgraduate	yes	yes

*Inpatient clinic Immanuel Hospital since 2001 (1989–2001 Moabit Hospital).

**Table 2:** Recommendations for junior faculty.

Personal skills	Professional development	Networking
(i) Enjoy the work (ii) Know your skills (iii) Know your interests (iv) Have a vision (v) Keep the vision	(i) Learn about existing models of integration (ii) Be a great M.D./Ph.D. (iii) Build a research team (iv) Build up a research program step-by-step	(i) Work with funders (ii) Make allies with colleagues at conventional medicine departments (iii) Align yourself with existing strengths of a hospital/research center
